# Two-year immune effect differences between the 0–1–2-month and 0–1–6-month HBV vaccination schedule in adults

**DOI:** 10.1186/s12879-022-07151-6

**Published:** 2022-02-18

**Authors:** Juan Wang, Chang-Hai Liu, Yuanji Ma, Xia Zhu, Liru Luo, Yulin Ji, Hong Tang

**Affiliations:** 1 Center of Infectious Diseases, West China Hospital of Sichuan University, No.37 Guoxue Alley, Chengdu, 610041 Sichuan Province China; 2 Division of Infectious Diseases, State Key Laboratory of Biotherapy and Center of Infectious Disease, West China Hospital, Sichuan University, Chengdu, China; 3 Department of Respiratory and Critical Care Medicine, West China Hospital of Sichuan University, Chengdu, China

**Keywords:** Hepatitis B vaccination, Anti-HBs level, Long-term immune effect

## Abstract

**Background:**

The short-term 0–1–2-month hepatitis B virus (HBV) vaccination schedule was previously implemented in the adult population; however, its long-term immune effect remains unclear. The present study aimed to investigate (1) the 2-month and 2-year immune effects of HBV vaccination and (2) the compliance rate between the 0–1–2-month and 0–1–6-month vaccination schedules in adults.

**Method:**

A total of 1281 subjects tested for hepatitis B surface antigen HBsAg(−) and hepatitis B surface antibody (anti-HBs)(−) were recruited. Participants from two distant counties were inoculated with the hepatitis B yeast vaccine at 10 µg per dose, with vaccination schedules of 0, 1, and 2 months (n = 606) and 0, 1, and 6 months (n = 675); sequential follow-up was performed at 2 months and 2 years after the 3rd injection.

**Results:**

There were no significant differences in the anti-HBs seroconversion rates between the those in the 0–1–2-month and 0–1–6-month vaccination schedule groups at 2 months (91.96% vs. 89.42%, p = 0.229) and 2 years (81.06% vs. 77.14%, p = 0.217). The quantitative anti-HBs level in those in the 0–1–2-month vaccination schedule group was not different from that in those in the 0–1–6-month vaccination schedule group at 2 months (anti-HBs_1_) (342.12 ± 378.42 mIU/ml vs. 392.38 ± 391.96 mIU/ml, p = 0.062), but it was higher at 2 years (anti-HBs_2_) (198.37 ± 286.44 mIU/ml vs. 155.65 ± 271.73 mIU/ml, p = 0.048). According to the subgroup analysis, the 0–1–2-month vaccination schedule induced better maintenance (p = 0.041) and longer reinforcement (p = 0.019) than the 0–1–6 vaccination schedule. The 0–1–2-month vaccination schedule group also had a higher 3rd injection completion rate (89.49% vs. 84.49%, p = 0.010).

**Conclusion:**

The 0–1–2-month vaccination schedule was associated with a similar short-term immune effect and might induce better long-term immune memory and a higher completion rate in the adult population.

*Trial registration* None

**Supplementary Information:**

The online version contains supplementary material available at 10.1186/s12879-022-07151-6.

## Background

The global seroprevalence of hepatitis B surface antigen (HBsAg) was estimated to be 3.9% in 2016 [1]. A modelling study [2] estimated the hepatitis B surface antigen (HBsAg) prevalence in China to be 6.1% [1], and another study reported that the prevalence of chronic hepatitis B virus (HBV) infections in 2018 was more than 80 million [3]. The World Health Organization (WHO) estimates that more than 658,000 individuals die annually from hepatitis B virus (HBV)-related complications, such as fulminant hepatitis, cirrhosis, and liver cancer [4]. Universal infantile HBV vaccination under the national immunization program has achieved great success in preventing and controlling HBV infection in the past 20 years. The HBsAg positive rate in mainland China decreased from 14.0% in 1957–89 to 5.4% in 1990–2013 [5]. In adults, the immunized population had a much lower seroprevalence of HBsAg than the unimmunized population [6]. Therefore, HBV immunization in adults should be recommended [2].

HBV immunization at 0, 1 and 6 months (0–1–6-month vaccination schedule) has been recommended by the World Health Organization (WHO) and US Centers for Disease Control and Prevention, as well as the Chinese National Guidelines on chronic HBV prevention and treatment (2015). However, the WHO-recommended 0–1–6-month vaccination schedule often leads to a lower vaccination completion rate in adults [7–9]. In China, there is a special population called the floating population (so-called migrant workers in other countries), comprising approximately 230 million people per year. Generally, these people leave their hometowns to find jobs in other cities and change jobs frequently. The floating population in China has an increased risk of sexual transmission of HBV due to a lower education level, a lower economic income level, younger age, and multiple sexual partners [10]. Therefore, previous studies proposed a vaccination schedule at months 0, 1, and 2 month (0–1–2-month vaccination schedule), with comparable short-term safety and immunogenicity [11, 12]. Moreover, shortening the vaccine schedule time can effectively increase the completion rate of vaccination and even stimulate earlier and faster hepatitis B surface antibody (anti-HBs) production [11, 12]. Accelerated immunization schedules (0–1–2 months, 0–1–3 months and 0–7–21 days, etc.) have been verified to have a similar short-term immune effect as longer schedules and improve the completion rate in the general population [13], injection drug users [14, 15] and adults who refuse to receive a second or third dose owing to occupational reasons [16, 17]. However, antibody maintenance via immune memory is even more crucial than antibody production in protecting patients from HBV infection. Therefore, comparative results for the most effective vaccination schedule could not be obtained from previous short-term follow-up studies.

The long-term immune effect of the accelerated vaccination schedule remains unclear due to a lack of evidence [11, 18]. Ren et al. recently reported the same positive seroprotection rate and quantitative anti-HBs level between a 0–1–3-month vaccination schedule and a 0–1–6-month schedule at 8 years after vaccination [17]. However, the author also declared high loss to follow-up (771 participants enrolled to 242 in the final follow-up, 529 participants were lost to follow-up) caused by the floating population and a relatively small sample size, potentially influencing the reliability, as limitations. We proposed a prospective interventional study in two comparable towns to investigate the short-term and long-term immune effects, as well as the completion rates, between an accelerated vaccination schedule (0–1–2-month schedule) and the standard vaccination schedule (0–1–6-month schedule).

## Methods

### Study design

This was a prospective study to explore the response of different HBV vaccination schedules in both HBsAg(−) and anti-HBs(−) adults, which were confirmed by using ELISA test (InTec PRODUCT, Xiamen, China) in township hospital. Randomization was not possible due to the different 3rd injection times (participants from small towns would be aware of the different schedules). Thus, two similar demographic and comparable towns (similar economic levels, dietary habits, social factors, etc.) Participants from Jinfeng town and Longmen town of Mianyang city were recruited. Participants 18 to 59 years old were enrolled. Participants with HIV coinfection and active HBV infection were excluded. The study was conducted from 1 June 2013 to 1 December 2017. The study protocol conformed to the ethical guidelines of the 1975 Declaration of Helsinki. The Institutional Review Board Committee of West China Hospital of Sichuan University approved the study protocol. The study was performed according to the ethical guidelines expressed in the Declaration of Helsinki and the International Conference on Harmonization Guidelines for Good Clinical Practice. Informed consent was obtained from all subjects.

This study was supported by the National Scientific and Technological Major Project for Infectious Diseases Control in China (Grant number 2018ZX10715-003) and The Science and Technology Project of The Health Planning Committee of Sichuan (Grant number 16PG280). The funding body was in charge of the design of the study and collection, analysis, and interpretation of data.

Appropriate training was provided to the research staff from the leading investigators. Standardized, questionnaire-based, face-to-face interviews were performed after obtaining written consent. The questionnaire collected information about sex, age, height and weight. Each subject was confirmed by screening their identification card and taking a photo before each vaccine injection. After that, they were offered vaccination through the regular service of township hospitals, where trained medical staff administered vaccinations by intramuscular injection. Vaccinations with 10 µg per dose of hepatitis B yeast vaccine (Hualan Biological Vaccine Company, Chengdu, China) were provided for the 0–1–2-month vaccination schedule in Jinfeng and the 0–1–6-month vaccination schedule in Longmen. This study was reported following the STrengthening the Reporting of OBservational studies in Epidemiology (Additional file 5).

### Follow-up

The 1st and 2nd follow-up visits were conducted at 2 months and 2 years after administration of the third vaccine injection, respectively. The follow-up time for each subject was shared by telephone call 3 weeks before the visit. Each subject was confirmed by screening their identification card and taking a photo. Every screening card was individualized with the follow-up time of each participant as a memorandum. Both schedules were consistent with the community standard of care at the time.

### Serum assay and blood sample tests

Serum samples were collected and tested at each follow-up visit in local health stations and community clinics. The anti-HBs levels at 2 months (anti-HBs_1_) and 2 years (anti-HBs_2_) after the 3rd injection were analysed to assess vaccine response. The serum samples were tested for the quantification of HBsAg and anti-HBs by electrochemiluminescence immunoassay (Abbott i2000SR, USA) in West China Hospital. An HBsAg level < 0.05 mIU/ml was defined as negative. Anti-HBs levels below 10 mIU/ml, 10–100 mIU/ml, 100–1000 mIU/ml and above 1000 mIU/ml were defined as no response, low response, normal response and high response, respectively [19]. Because an anti-HBs ratio less than 0.05 mIU/ml was unable to be detected and exceeding 1000 mIU/ml was excluded from further dilution tests, values of 0 mIU/ml and 1000 mIU/ml were assigned to these subjects for quantitative analysis of anti-HBs, for reference to the previous anti-HBs geometric mean concentration (GMC) test [12, 20].

To explore the reason for different maintenance of anti-HBs levels between those receiving the two vaccination schedules, the subjects were divided into four different clinical scenario groups: (1) “well production and good maintenance”, indicated by anti-HBs_1_ (at 2 months) (+) and anti-HBs2 (at 2 years) (+); (2) “well production and poor maintenance”, indicated by anti-HBs_1_ (+) and anti-HBs_2_ (−); (3) “persistent non-production”, indicated by anti-HBs1 (−) and anti-HBs_2_ (−); and (4) “delayed production”, indicated by anti-HBs_1_ (−) and anti-HBs_2_ (+). Furthermore, the “well production and good maintenance” group was further divided into four subgroups: (1) “high production and good maintenance”, indicated by high anti-HBs_1_ (> 100 mIU/ml) and high anti-HBs_2_ (> 100 mIU/ml); (2) “well production and fast decrease”, indicated by high anti-HBs_1_ (> 100 mIU/ml) and low anti-HBs_2_ (10–100 mIU/ml); (3) “relative low production”, indicated by low anti-HBs_1_ (10–100 mIU/ml) and low anti-HBs_2_ (10–100 mIU/ml); and 4) “delayed reinforcement”, indicated by low anti-HBs_1_ (10–100 mIU/ml) and high anti-HBs_2_ (> 100 mIU/ml).

### Statistical analysis

Mean and prevalence values of baseline characteristics were calculated. Data are reported as the mean ± standard deviation (SD) for normally distributed data and the median (interquartile range, IQR) for nonnormally distributed continuous variables (when the sample size > 40, the mean ± SD was used to represent data), while the frequency was used for discrete variables. In the univariate comparisons, we used Student’s t-test and ANOVA with Bonferroni adjustments for continuous variables and the Chi-square test or Fisher’s exact test for qualitative variables. Nonparametric alternatives (Mann–Whitney U and Kruskal–Wallis tests) were used for variables with nonnormal distributions. Logistic regression models were used to estimate adjusted odds ratios (aORs) with our principal outcome of full-time completion rate and 3rd injection rate. Covariates were selected for analysis according to their biologically plausible potential to act as confounders or predictors for each outcome. The potential predictors at baseline were as follows: age, sex, body mass index (BMI), previous hypertension, previous type 2 diabetes mellitus (T2DM), and abnormal alanine aminotransferase (ALT) level. The collinearity between factors included in the multivariable analyses was checked by using variance inflation factor (VIF) and tolerance (1/VIF) values. Variables with very high VIF values indicating possible redundancy entered into different multivariable models. Statistic method for adjusting the quantitative bias from the T.L. Lash’s textbook were performed to minimize the bias [21, 22]. All adjusted odds ratios from multivariant analysis, was further adjusted by accounting loss-to-follow-up bias. All statistical analyses and figures were performed in SPSS (version 20) and PRISMA (version 8). A p value < 0.05 was considered statistically significant.

## Results

### Characteristics of the included population and follow-up data

A total of 1281 subjects with both HBsAg (−) and anti-HBs (−) were enrolled. Of them, 606 subjects from Jinfeng were assigned to the 0–1–2-month vaccination schedule group, while the other 675 subjects from Longmen were assigned to the 0–1–6-month vaccination schedule group (Additional file 1: Fig. S1). In total, 511 subjects in the 0–1–2-month vaccination schedule group and 550 subjects in the 0–1–6-month vaccination schedule group completed all three injections. The baseline characteristics of the included participants are shown in Table 1. In brief, age (37.74 ± 12.64 vs. 38.80 ± 12.13, p = 0.129), sex (male: 45.37% vs. 54.63%, p = 0.256) and BMI (23.02 ± 3.54 vs. 22.85 ± 3.85, p = 0.422) were similar between the 0–1–2-month and 0–1–6-month vaccination schedule groups (Table 1).

**Table 1:** Baseline characteristics of included participants

Variables	0–1–6-month vaccination (n = 675)	0–1–2-month vaccination (n = 606)	p value
Age, years	38.80 ± 12.13	37.74 ± 12.64	0.129
Sex Male	288 (54.63%)	239 (45.37%)	0.256
Female	387 (51.33%)	367 (48.77%)	
BMI, kg/m^2^	22.85 ± 3.85	23.02 ± 3.54	0.422
Waistline, cm	76.20 ± 11.04	77.45 ± 9.31	0.030
ALT	29.97 ± 27.90	34.46 ± 32.56	0.008
Abnormal ALT	116 (45.13%)	141 (54.93%)	0.007

*BMI* body mass index, *ALT* alanine aminotransferase

A total of 621 participants completed both 2-month and 2-year follow-up visits. The information obtained from phone calling indicated that the reasons for not finishing the three injections or not showing up to the follow-up were mainly due to working schedules or migrant work in distant locations. The distribution was equally reassessed since almost half of the participants were lost to follow-up (Additional file 2: Table S1).

### Comparison of the short- and long-term immune effects of the two vaccination schedules

**Fig. 1:**
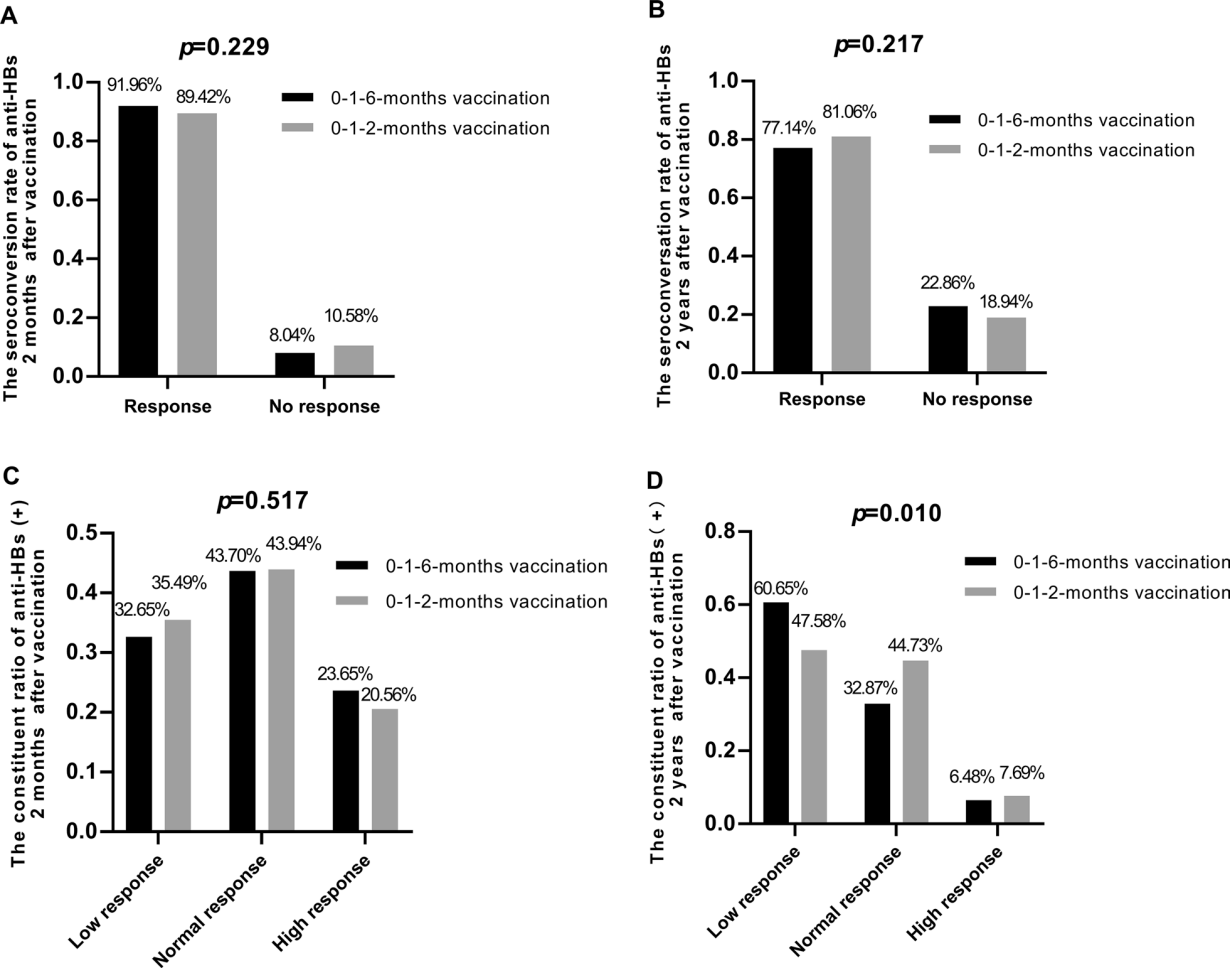
Short- and long-term anti-HBs seroconversion. The rate of anti-HBs seroconversion at 2 months (**A**) and 2 years (**B**) after vaccination; the proportion of anti-HBs levels according to no response (10–100 mIU/ml), low response (100–1000 mIU/ml) and high response (≥ 1000 mIU/ml) at 2 months (**C**) and 2 years (**D**) after vaccination

**Fig. 2:**
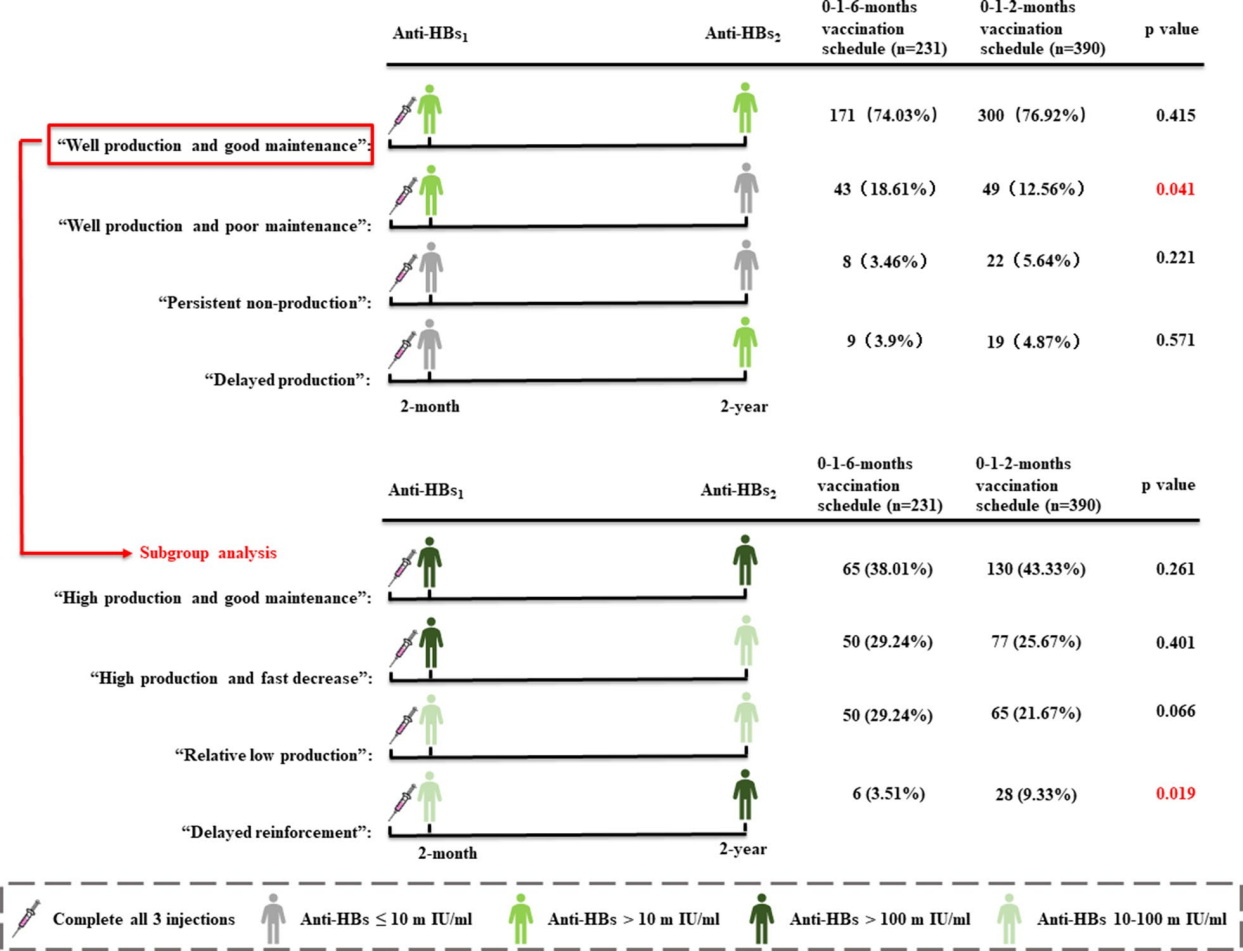
Comparisons of the different immune memories between the different vaccination schedules groups, indicated by 2-month and 2-year anti-HBs levels. The 621 participants who completed both the 2-month and 2-year follow-up were included in the first part of the analysis; 471 participants in the “well production and good maintenance” group in the first part of the analysis, with anti-HBs1(+) and anti-HBs2(+), were included in the second part of analysis and were further divided into four different subgroups. Anti-HBs1, hepatitis B antibody at 2 months after three vaccine injections; Anti-HBs2, hepatitis B antibody at 2 years after three vaccine injections

1. No difference was found in anti-HBs seroconversion rate between the two vaccination schedule groups.
  Anti-HBs seroconversion rates were not significantly different between the 0–1–2-month and 0–1–6-month vaccination schedule groups for short-term seroconversion (at 2 months) (89.42% vs. 91.96%, p = 0.229) (Fig. 1A) or long-term seroconversion (81.06% vs. 77.14%, p = 0.217) (Fig. 1B). Therefore, a similar immune effect was obtained between the two vaccination schedules, and even earlier protection was gained at 2 months as opposed to 6th months in the 0–1–6-month vaccination schedule.
2. Those under the 0–1–2-month vaccination schedule showed a higher anti-HBs level at the 2-year follow-up.
  The quantitative result of anti-HBs in the 0–1–2-month vaccination schedule group was not different at the 2-month follow-up (342.12 ± 378.42 mIU/ml vs. 392.38 ± 391.96 mIU/ml, p = 0.062), but it was higher at the 2-year follow-up (198.37 ± 286.44 mIU/ml vs. 155.65 ± 271.73 mIU/ml, p = 0.048) than that in the 0–1–6-month vaccination schedule group (Table 2). Furthermore, among the subjects with successful seroconversion (anti-HBs_1_ > 10 mIU/ml), no difference was found in the proportion of low responses (10 mIU/ml < anti-HBs_1_ < 100 mIU/ml), normal responses (100 mIU/ml < anti-HBs_1_ < 1000 mIU/ml) and high responses (anti-HBs_1_ > 1000 mIU/ml) between the 0–1–2-month and 0–1–6-month vaccination schedule groups at the 2-month follow-up (p for ANOVA = 0.517) (Fig. 1C). However, the 0–1–2-months vaccination schedule induced better maintenance of anti-HBs at the 2-year follow up, with a higher proportion of normal responses (44.73% vs. 32.87%) and high responses (7.69% vs. 6.48%) and a lower proportion of low responses (7.69 vs. 6.48%) (p for ANOVA = 0.010) (Fig. 1D).
3. The 0–1–2-month vaccination schedule was associated with better maintenance and delayed reinforcement.
  To explore the reason for the different maintenance levels of anti-HBs between the two vaccination schedule groups, the subjects were divided into four different clinical scenarios (detailed in “Methods” section). We found that the 0–1–2-month vaccination schedule group had a lower proportion of people in the “well production and poor maintenance” group than the 0–1–6-month vaccination group (12.56% vs. 18.61%, p = 0.041), suggesting that the 0–1–2-month vaccination schedule induced better maintenance than the 0–1–6 vaccination schedule (Fig. 2). The “well production and good maintenance” group was further divided into four subgroups by the qualitative level of anti-HBs. The results showed that the proportion of the 4th subgroup (low anti-HBs_1_ and high anti-HBs_2_) in the 0–1–2-month vaccination schedule group was higher than that in the 0–1–6-month vaccination schedule group (9.33% vs. 3.51%, p = 0.019), suggesting that the 0–1–2-month vaccination schedule possibly induced “delayed reinforcement” antibody production, as patients had low anti-HBs initially but high anti-HBs at 2 years (Fig. 2).

**Table 2:** Short-term and long-term comparison of quantitative anti-HBs between 0–1–2-months and 0–1–6-months vaccination schedules

Variables	0–1–6-months vaccination (n = 231)	0–1–2-months vaccination (n = 390)	p value
	Mean ± SD	Mean ± SD	
Anti-HBs_1_ (mIU/ml)	392.38 ± 391.96	342.12 ± 378.42	0.062
Anti-HBs_2_ (mIU/ml)	155.65 ± 271.73	198.37 ± 286.44	0.048

Anti-HBs_1_, hepatitis B antibody at 2 months after three vaccination injections; Anti-HBs_2_, hepatitis B antibody at 2 years after three vaccination injections

### Factors associated with anti-HBs seroconversion

In the multivariate analysis, no significant difference in the anti-HBs seroconversion rate was found between the 0–1–2-month and 0–1–6-month vaccination schedule groups at either 2 months (OR 0.73, 95% CI 0.46–1.18, p = 0.211) or 2 years (OR 1.27, 95% CI 0.85–1.90, p = 0.241) (Table 3). In addition, younger age (OR 0.97, 95% CI 0.95–0.99, p = 0.007), lower BMI (OR 0.86, 95% CI 0.81–0.92, p < 0.001) and lower anti-HBc level (OR 0.88, 95% CI 0.83–0.94, p < 0.001) were significantly associated with anti-HBs seroconversion at the 2-month follow-up. Only a lower BMI (OR 0.91, 95% CI 0.86–0.96, p = 0.002) was significantly associated with anti-HBs seroconversion at 2 years. Moreover, after adjusting loss-follow-up bias by T.L. Lash statistic method, the anti-HBs seroconversion rate was found between the 0–1–2-month and 0–1–6-month vaccination schedule groups at either 2 months (OR 0.63, 95% CI 0.36–2.08, p = 0.535) or 2 years (OR 0.87, 95% CI 0.55–1.90, p = 0.347).

**Table 3:** Univariate analysis to identify variables associated with short-term and long-term anti-HBs seroconversion rate

Variables	Anti-HBs seroconversion at 2 months		Anti-HBs seroconversion at 2 years	
	Odds ratio (95% CI)	p value	Odds ratio (95% CI)	p value
Age, years	0.97 (0.95–0.99)	0.007	0.98 (0.97–1.00)	0.165
Male sex	1.17 (0.73–1.90)	0.503	0.73 (0.48–1.11)	0.147
BMI	0.86 (0.81–0.92)	< 0.001	0.91 (0.86–0.96)	0.002
Anti-HBc	0.88 (0.83–0.94)	< 0.001	1.04 (0.98–1.09)	0.148
ALT	0.99 (0.99–1.00)	0.282	1.00 (0.99–1.00)	0.910
0–1–2-month vs. 0–1–6-month (reference) vaccination schedule	0.73 (0.46–1.18)	0.211	1.27 (0.85–1.90)	0.241

### Comparison of vaccination completion rates

Since the 3rd injection completion rate at the 2nd or 6th month was the main difference between the 0–1–2-month and 0–1–6-month vaccination schedule groups, we further analysed the 3rd injection completion rate. The 3rd injection completion rate in the 0–1–2-month vaccination schedule group was higher than that in the 0–1–6-month vaccination schedule group (89.49% vs. 84.49%, p = 0.010) (Additional file 3: Fig. S2) Moreover, after adjustment for age and BMI, the 3rd injection completion rate was significantly increased in the 0–1–2-month vaccination schedule group (OR 1.69, 95% CI 1.19–2.39, p = 0.003) compared to the 0–1–6-month vaccination schedule group as a reference (Table 4). This OR (OR 1.89, 95% CI 1.23–2.89, p = 0.012) of 3rd injection completion was kept significant after using the T.L. Lash statistic method for adjusting loss-follow-up.

**Table 4:** Univariate and multivariant analysis to identify variables associated with 3rd-time injection completion rate

	Successful 3rd-time injection completion rate			
	Univariant aOR (95% CI)	p value	Multivariant aOR (95% CI)	p value
Age	1.04 (1.03–1.06)	< 0.001	1.04 (1.03–1.06)	< 0.001
Sex (Female)	1.38 (0.99–1.93)	0.053		
BMI	1.06 (1.01–1.11)	0.012	0.99 (0.94–1.04)	0.092
Hypertension	7.25 (0.35–35.98)	0.146		
T2DM	7.10 (0.97–51.91)	0.530		
Abnormal ALT	1.55 (0.974–2.47)	0.065		
Vaccination type (0–1–2-months vs. 0–1–6-months)	1.56 (1.112–2.20)	0.010	1.68 (1.19–2.39)	0.003

*BMI* body mass index, *T2DM* type 2 diabetes mellitus, *aORs* adjusted odds ratios, *ALT* alanine aminotransferase

## Discussion

In the present study, we found that short- and long-term anti-HBs seroconversion rates were not different between the 0–1–2-month and 0–1–6-month vaccination schedule groups. However, the 0–1–2-month vaccination schedule group showed a higher anti-HBs level at the 2-year follow-up, suggesting better maintenance and delayed reinforcement, than the 0–1–6-month vaccination schedule group. In the multivariate analysis, no significant difference in the anti-HBs seroconversion rate was found between the 0–1–2-month and 0–1–6-month vaccination schedule groups at 2 months and 2 years. However, the 0–1–2-month vaccination group had a significantly higher 3rd injection completion rate. Therefore, in conclusion, the 0–1–2-month vaccination schedule can induce similar short-term immune effects and might induce better long-term immune memory and a higher completion rate in the adult population.

In a recent systematic review, different vaccination schedules induced similar short-term immune effects, with anti-HBs concentrations ≥ 10 mIU/ml in approximately 65.0–85.0% of those in the 0–1–2-month schedule group, approximately 77.0–90.8% in those in the 0–7–21-day schedule group, 87.0% in those in the 0–2–6-week schedule group, and approximately 79.0% in those in the 0–14–28-day schedule group [23]. Ren et al [17] also reported that an accelerated schedule (0–1–3 months) and the standard schedule (0–1–6 months) enhanced long-term immune memory (8 years later) in comparison to the 0–1–12 months schedule, and we found a similar long-term immune effect in the present study (2 years later). Regarding the short-term immune effect, we speculate that the different short-term immune effects may be explained by the fact that the measurements were taken at different times (4 months for “0–1–3-month”, 7 months for “0–1–6-month” and 13 months for “0–1–12-month” schedules) since the 6-month and 12-month injections serve as booster doses, which is known to increase the seroconversion rate [23]. Regarding the long-term immune effect, we further explored whether prolonged immune memory with a shorter vaccination interval of 0–1–3 months was induced by “better maintenance” and “delayed reinforcement” in the present study. Thus, the shorter interval of the vaccination schedule might provide a stronger response by the immune system, resulting in a higher anti-HBs level at 2 years after three injections. However, since there was substantial loss-to-follow-up at the 2nd-year assessment, future studies should re-evaluate the present result.

The developing immune system in children is different from the mature immune system in adults; therefore, vaccination schedules could be modified according to specific populations. Cesare Belloni [24] reported that in children, the 0–1–6-month vaccination schedule presented a higher percentage of seroconversion than the 0–1–3-month vaccination schedule but also pointed out that the reduced response after the 0–1–3-month vaccination schedule was mainly due to the relative immaturity of the immune system in younger infants [25]. Similarly, for bacterial conjugate vaccines, it is recommended to administer only one dose for initial vaccination and a booster immunization in adults, which is sufficient for adults to produce memory B cells and to maintain the antibody due to the mature immune system [26]. Thus, the decision of a shorter or longer period of vaccination injection was dependent on the mature or immature status of the host immune system. The immature immune system in children responded differently at 0 months, 1 month and 6 months since the system was still developing; thus, the 3rd injection at 6 months in children could induce a better response due to a relatively more mature immune system at 6 months than that at 3 months or earlier. However, the mature immune system in adults responded similarly at 0 months, 1 month, 3 months and 6 months; thus, a shorter period could result in a higher completion rate.

Our results indicated that the 0–1–2-month vaccination schedule was associated with a better completion rate than the 0–1–6-month vaccination schedule. However, a previous study reported that a 0–1–12-month vaccination schedule may be more suitable for the floating population and that a 0–1–6-month schedule is recommended for the fixed population; both of these schedules had better completion rates than the 0–1–3-month schedule [7]. The floating population of China is characterized by changing jobs frequently and returning home annually to celebrate the Spring Festival and rest after 11 months of work. The different results between the present and previous studies could be explained by calculating the 3rd injection rather than the completion time of injections since the 1st and 2nd injections were the same at 0 and 1 months.

There were limitations in the present study. First, some patients were lost to follow-up at the first and second follow-up visits. Nevertheless, loss to follow-up was inevitable because the secondary outcome of the present study was designed to investigate the completion rate in a floating population. Moreover, we reassessed the baseline characteristics of the followed patients, resulting in equal distribution between the two groups (Additional file 2: Table S1). Adjusted odds ratios from statistic method provided by T.L. Lash were performed to minimize the loss-to-follow-up bias. Second, information about the participants’ characteristics (including smoking, alcohol consumption, diabetes, wife/husband HBV status, people sharing home, drug addictive history and etc.) was not recorded (those with HIV coinfection and active HBV were excluded), which might affect the antibody response after hepatitis B vaccination. There was small part of included subject with elevated ALT levels in the baseline, which might possibly relate to HCV or fatty liver or other kind of liver disease. Nevertheless, the abnormal ALT was not significant influence either the Anti-HBs seroconversion at 2 months nor at 2 years, as well as the 3rd-time injection completion rate in two multivariant analysis. Future study could add the HCV and fatty liver status to re-evaluate the present concept. In addition, the included participants of present resent study was with both HBsAg(−) and anti-HBs(−) adults which tested by ELISA at baseline, but without information of anti-HBc; the future study should address this limitation. Last, a standardized time point for the measurement of anti-HBs levels was used to enhance the comparability of the immune response between different studies; although we found better maintenance and delayed reinforcement of anti-HBs with the 0–1–2-month vaccination schedule, the insufficient observation time points within 2 years limited us to exploring only the anti-HBs production tendency.

## Conclusion

The 0–1–2-month vaccination schedule obtained a similar short-term immune effect and might induce better long-term immune memory and a higher completion rate in the adult population.

## Supplementary Information

**Additional file 1: Figure S1.** Study flow diagram.

**Additional file 2: Table S1.** Characteristics of the population finished vaccination.

**Additional file 3: Figure S2.** Comparison of vaccination 3rd time completion rates in the different vaccination schedule groups.

**Additional file 4.** Additional data: raw data (2021.01.13) (englished version).

**Additional file 5.** Checklist for the appropriate reporting statement: STROBE.

## Data Availability

Additional file 4 is included. All data generated or analysed during this study are included in this published article and its Additional information files.

## Abbreviations

HBV: Hepatitis B virus
WHO: World Health Organization
HBsAg: Hepatitis B surface antigen
anti-HBs: Hepatitis B surface antibody
BMI: Body mass index
anti-HBs_1_: The anti-HBs level at 2 months after 3rd injection
anti-HBs_2_: Anti-HBs level at 2 years after the 3rd injection
aOR: Adjusted odds ratio
T2DM: Type 2 diabetes mellitus
ALT: Alanine aminotransferase
CI: Confidence interval
