# Supplementary figures and images for: Two-year immune effect differences between the 0–1–2-month and 0–1–6-month HBV vaccination schedule in adults
Source: BMC Infect Dis. 2022 Feb 18;22:159. doi: 10.1186/s12879-022-07151-6 (PMC8855546; doi:10.1186/s12879-022-07151-6)

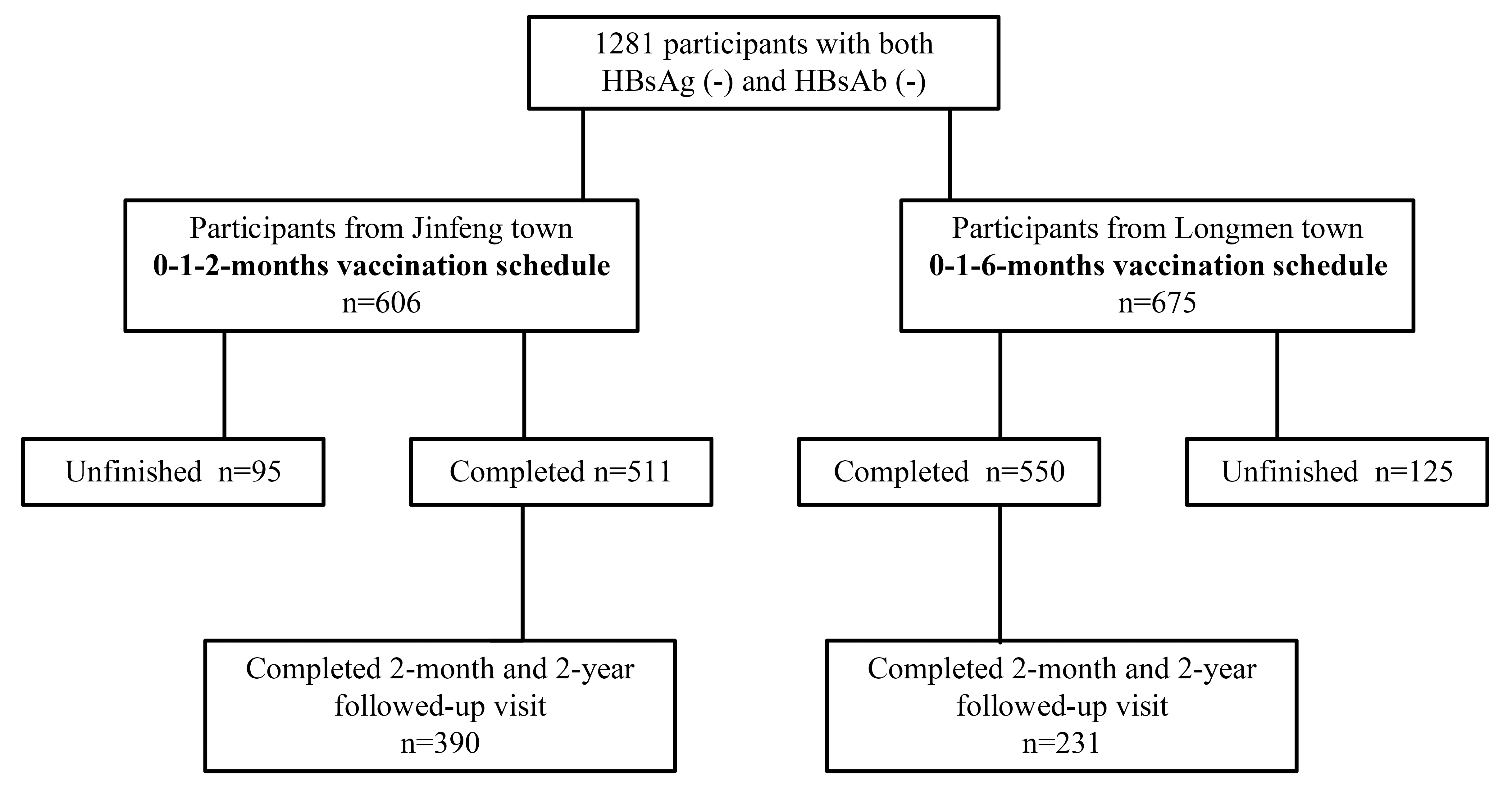

Supplement: Supplementary file 1 — Additional file 1: Figure S1. Study flow diagram. [file 12879_2022_7151_MOESM1_ESM.tif]

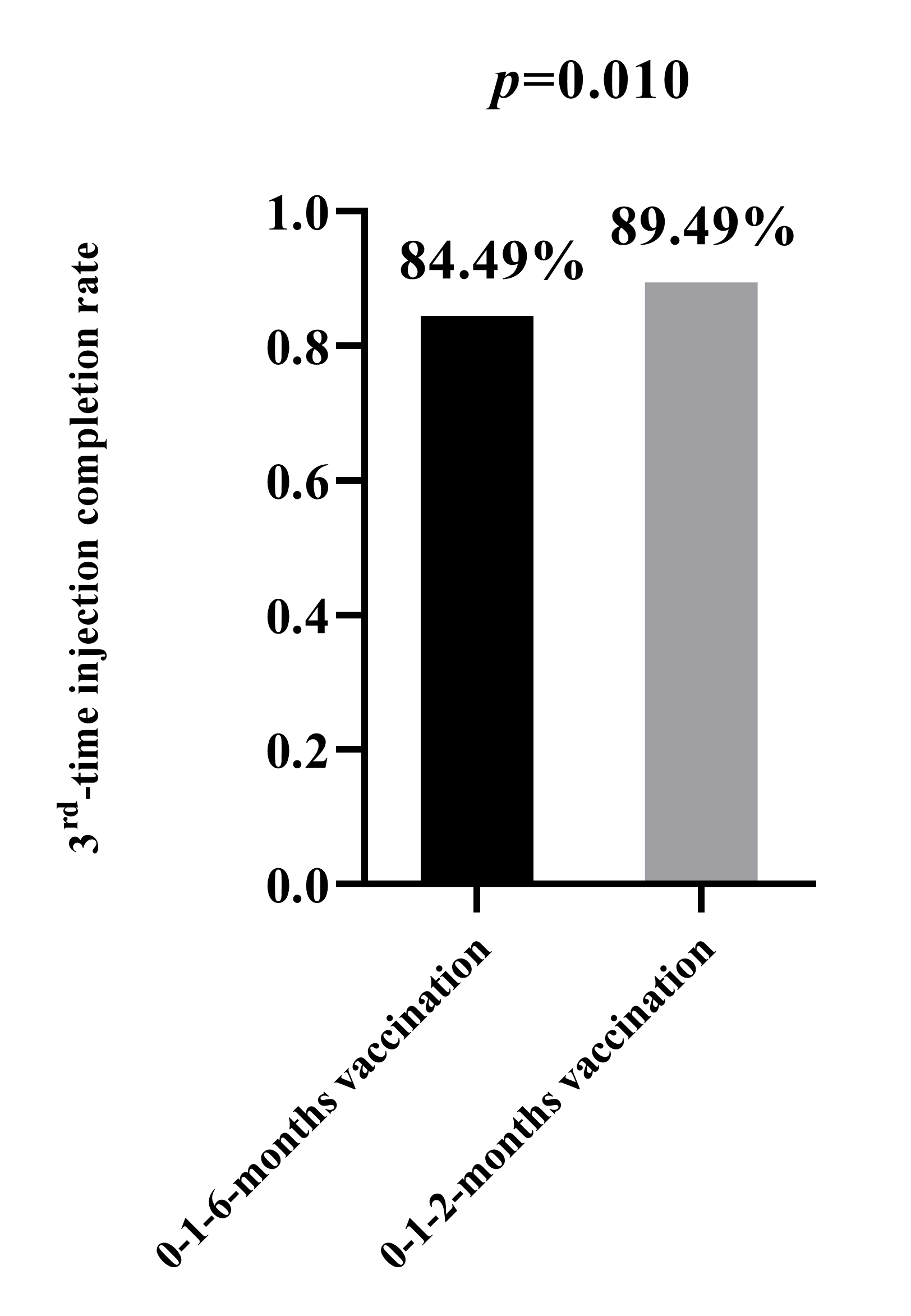

Supplement: Supplementary file 3 — Additional file 3: Figure S2. Comparison of vaccination 3rd time completion rates in the different vaccination schedule groups. [file 12879_2022_7151_MOESM3_ESM.tif]
