# Supplementary material for: Two-year immune effect differences between the 0–1–2-month and 0–1–6-month HBV vaccination schedule in adults
Source: BMC Infect Dis. 2022 Feb 18;22:159. doi: 10.1186/s12879-022-07151-6 (PMC8855546; doi:10.1186/s12879-022-07151-6)
Supplement: Supplementary file 2 — Additional file 2: Table S1. Characteristics of the population finished vaccination. [file 12879_2022_7151_MOESM2_ESM.docx]

**Table S1 Characteristics of the population finished vaccination**

|  | 0-1-6-month vaccination | 0-1-2-month vaccination | p value (1) between 0-1-6-month vaccination and 0-1-2-month vaccination | p value (2) between baseline and who finished vaccination |
| --- | --- | --- | --- | --- |
| Variables | Baseline (n=675) | Baseline (n=606) |  |  |
| Age, years | 38.80 ± 12.13 | 37.74 ± 12.64 | 0.129 |  |
| Sex Male | 288 (54.63%) | 239 (45.37%) | 0.256 |  |
| Female | 387 (51.33%) | 367 (48.77%) |  |  |
| BMI, kg/m^2^ | 22.85 ± 3.85 | 23.02 ± 3.54 | 0.422 |  |
| Waistline, cm | 76.20 ± 11.04 | 77.45 ± 9.31 | 0.030 |  |
| ALT | 29.97 ± 27.90 | 34.46 ± 32.56 | 0.008 |  |
|  | Finished vaccination (n=472) | Finished vaccination (n=440） |  |  |
| Age, years | 40.81 ± 11.5 | 39.5 ± 12.19 | 0.096 | 0.301 |
| Sex Male | 193(40.89%) | 167(37.95%) | 0.365 | 0.404 |
| Female | 279(59.11%) | 273(62.05%) |  |  |
| BMI, kg/m^2^ | 22.99±3.58 | 23.16±3.52 | 0.475 | 0.556 |
| Waistline, cm | 76.64±11.30 | 77.77±9.35 | 0.098 | 0.809 |
| ALT | 30.82±30.89 | 36.48±34.97 | 0.001 | 0.115 |

BMI, body mass index; SD, standard deviation. Two comparison were provided to ensure the distribution were equally.

P value (1): comparison between the 0-1-6-month vaccination schedule and 0-1-2-month vaccination schedule, separated from baseline and who finished vaccination.

P value (2): comparison between baseline and who finished vaccination, separated from between the 0-1-6-month vaccination schedule and 0-1-2-month vaccination schedule.
